# Protein ISGylation: a posttranslational modification with implications for malignant neoplasms

**DOI:** 10.37349/etat.2023.00162

**Published:** 2023-08-31

**Authors:** Angeles C. Tecalco-Cruz, Jesús Zepeda-Cervantes

**Affiliations:** Johannes-Gutenberg University of Mainz, Germany; ^1^Postgraduate in Genomic Sciences, Campus Del Valle, Autonomous University of Mexico City (UACM), CDMX 03100, Mexico; ^2^Department of Microbiology and Immunology, Faculty of Veterinary Medicine and Zootechnics, National Autonomous University of Mexico (UNAM), CDMX 04510, Mexico

**Keywords:** Interferon-stimulated gene 15, ISGylation, cancer

## Abstract

Interferon (IFN)-stimulated gene 15 (ISG15) is a member of the ubiquitin-like (UBL) protein family that can modify specific proteins via a catalytic process called ISGylation. This posttranslational modification can modulate the stability of the ISGylated proteins and protein-protein interactions. Some proteins modified by ISG15 have been identified in malignant neoplasms, suggesting the functional relevance of ISGylation in cancer. This review discusses the ISGylated proteins reported in malignant neoplasms that suggest the potential of ISG15 as a biomarker and therapeutic target in cancer.

## Introduction

**Figure 1:**
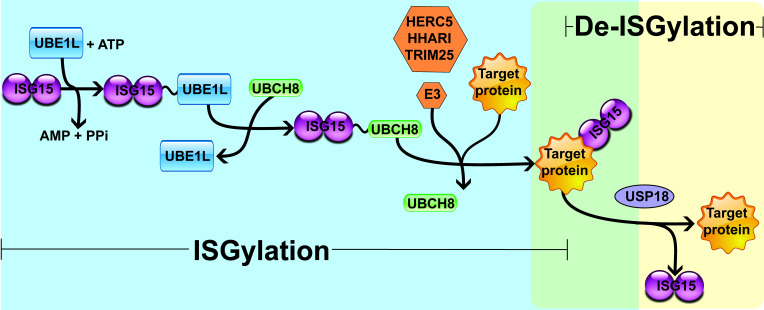
Enzymatic cascade reactions of ISGylation. UBE1L is associated with ISG15 by forming a thioester bond, activating ISG15 in an ATP-dependent manner. Next, E2 ligase (UBCH8) has a cysteine active site where ISG15 is transferred. Finally, E3 ligase (HERC5, HHARI, or TRIM25) catalyzes the covalent binding of ISG15 to its target protein. USP18 removes ISG15 from the ISGylated protein, mediating a de-ISGylation process. PPi: inorganic pyrophosphate

Interferon (IFN)-stimulated gene 15 (ISG15) is a 15 kDa protein composed of two ubiquitin-like (UBL) domains. A hinge sequence connects the N-terminal UBL domain to the C-terminal UBL domain, which has a motif containing lysine, arginine and glycine residues (LRLRGG) [1–4]. Through this sequence, ISG15 is covalently associated with its target proteins on lysine (Lys) residues by the sequential actions of the E1-activating enzyme (UBE1L), the E2-conjugating enzyme [ubiquitin-conjugating enzyme E2 L6 (UBCH8)], and the E3 ligases [HECT and RLD domain containing E3 ubiquitin protein ligase 5 (HERC5), ariadne RBR E3 ubiquitin protein ligase 1 (HHARI), and tripartite motif containing 25 (TRIM25)] [5–8]. This process is known as ISGylation and occurs in three steps similar to the protein ubiquitination process: (A) UBE1L mediates the formation of an adenosine triphosphate (ATP)-dependent thioester bond with ISG15; (B) ISG15 is transferred from UBE1L to UBCH8 through a transesterification reaction, forming a thioester bond between ISG15 and UBCH8; (C) from the ISG15-E2 enzyme complex, the E3 ligases promote the transfer and covalent attachment of ISG15 to the Lys residue of the target proteins. Hence, the E3 ligases HERC5, HHARI, and TRIM25 mediate the substrate specificity for ISGylation [5–8]. Protein ISGylation is regulated by a de-ISGylase enzyme named ubiquitin-specific peptidase 18 (USP18) that removes ISG15 from target proteins, reducing ISGylation and increasing free ISG15 levels (Figure 1) [9–11].

Interestingly, many proteins may be ISGylated by HERC5 in a co-translational manner since HERC5 is localized in the polysomes and associates with the 60S ribosomal subunit [12]. It has been proposed that ISGylation occurs in proteins that suffer premature translation termination and in misfolded proteins to remove them from functional proteins [13, 14]. Moreover, compared with other posttranslational modifications, only a few ISGylated proteins have been reported. Consequently, the E3 ligase and the Lys residue or residues where ISGylation occurs are known only for some ISGylated proteins. This modification has been shown to occur mainly as monoISGylation on one, two, or multiple Lys residues [15, 16]. Some ISGylated proteins within the cancer context are displayed in Table 1.

**Table 1:** Examples of ISGylation target proteins

**Protein**	**ISGylation sites**	**E3 ligase**	**Activity**	**Effect of ISGylation on stability or activity**	**Reference**
TRIM25	K117	TRIM25	E3 ISG15 ligase	Reduces the TRIM25 activity as an E3 ligase for ISG15	[17]
			E3 ubiquitin ligase	ND	
Filamin B	K2467	ND	Scaffold protein	Affects interactions, reducing MAPK and JNK signaling	[18]
PARK	K349 K369	HERC5	E3 ubiquitin ligase	Increases its E3 ubiquitin activity Increases its cytoprotective effect	[19]
ΔNp63α	K139 K324	ND	Pro-tumor	Reduces ΔNp63α activity and promotes tumor growth	[20]
BECN1	K117 K263 K265 K266	HERC5	Autophagy-associated protein	Inhibits autophagy and promotes antiviral responses	[15]
4EHP	K134 K222	HHARI	Translation repressor (cap-binding)	Increases the cap structure-binding activity Inhibits the translation of mRNAs	[21]
14-3-3σ	ND	TRIM25	Associated protein with oncogenic signaling	ND	[22]
14-3-3ζ	ND	ND	Oncogenic signaling	Affects the stability of 14-3-3ζ Loss of USP18 destabilizes 14-3-3ζ protein, repressing lung cancer metastasis	[23]
PCNA	K164 K168	TRIM25	DNA replication and repair	Terminates error-prone TLS Prevents excessive mutagenesis	[24]
p53	Multiple sites	HERC5	Tumor suppressor	Inactivates p53 tumor suppressor	[16]
				Facilitates degradation of misfolded p53 (via 20S proteasome)	[14]
	K291 K292	TRIM25		Increases the transcriptional activity of p53	[25]
HIF-1α	Multiple sites	HERC5	Transcription factor	Reduces HIF-1α levels Reduces HIF-1α-induced expression	[26]
β-catenin	ND	HERC5	Co-factor	Increases the degradation of β-catenin (ISGylation-dependent ubiquitination) in colon cancer cells	[27, 28]
FOXO3A	ND	ND	Transcription factor	Increases degradation of FOXO3A in human lung fibroblasts	[29]
PTEN	C-terminus	ND	Tumor suppressor (phosphatase)	Decreases the stability of PTEN, reducing its tumor suppressor activity, but USP18 stabilizes PTEN protein	[30]
EMD	K37	ND	Pro-tumor	Inhibits the EMD ubiquitination, increasing its stability and pro-tumor activity	[31]
YAP	K497	HERC5	Pro-tumor Co-factor	Reduces the degradation of YAP, increasing its pro-tumor activity	[32]
Ki-ras (GDI2)	Several sites	ND	Pro-tumor	Increases the endocytic recycling of the EGFR and sustained Akt signaling Breast cancer progression	[33]
OCT4	K284	ND	Transcription factor	Enhances the stability of OCT4 Promotes glioma cell stemness	[34]

ND: not determined; MAPK: mitogen-activated protein kinase; JNK: c-Jun N-terminal kinase; PARK: parkin; ΔNp63α: alternative splice variant of phosphoprotein 63; BECN1: beclin 1; 4EHP: eukaryotic translation initiation factor 4E homologous protein; 14-3-3σ: stratifin; PCNA: proliferating cell nuclear antigen; TLS: translesion DNA synthesis; p53: phosphoprotein 53; HIF-1α: hypoxia-inducible factor 1 subunit α; FOXO3A: forkhead box O3A; PTEN: phosphatase and tensin homolog; EMD: skeletal protein emerin; YAP: Yes-associated protein; EGFR: epidermal growth factor receptor; Akt: Akt kinase; Ki-ras: KRAS proto-oncogene, GTPase; GDI2: guanosine diphosphate (GDP) dissociation inhibitor 2; OCT4: POU class 5 homeobox 1 (also known as POU5F1)

## Molecular actions of ISGylation

**Figure 2:**
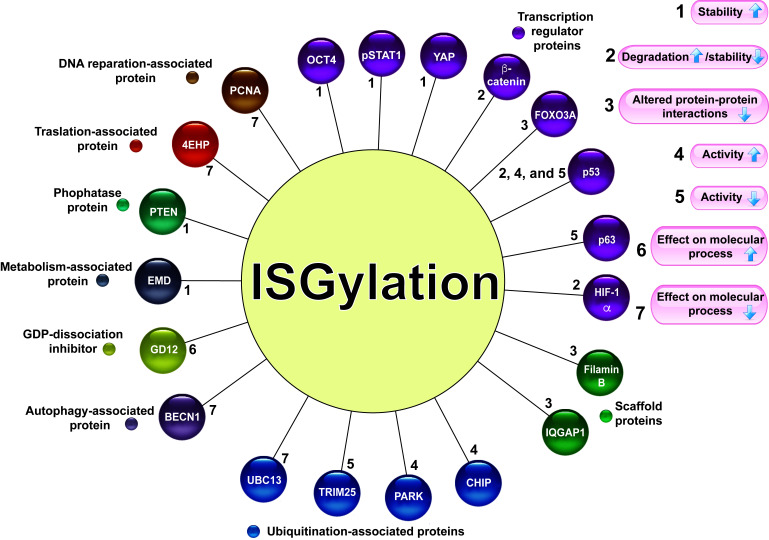
Target proteins for ISGylation. Several proteins associated with different molecular activities are modified by ISGylation (circles of different colors). The numbers 1–7 indicate the effect of ISGylation (the arrows indicate increase or decrease) on its target proteins. pSTAT1: phosphorylated signal transducer and activator of transcription 1 (STAT1); IQGAP1: IQ motif containing GTPase activating protein 1; UBC13: E2 ubiquitin-conjugating protein UBC13; CHIP: C-terminus of the Hsc70-interacting protein

ISGylation is a posttranslational modification related to changes in protein stability, increasing or decreasing protein levels, by competing with or promoting degradation via the ubiquitin-proteasome system (UPS) or lysosome-associated pathway. ISGylation can also modify the protein interaction pattern. The molecular actions associated with protein ISGylation are described in Figure 2.

### Effect of ISGylation on protein stability

The effect of ISGylation is related to increased protein stability by competing with or blocking ubiquitination and degradation via the UPS. For instance, IFN-stimulated cells treated with proteasome inhibitors enhance ISGylation levels [35]. In addition, when ISGylation levels are decreased by reducing the expression of *ISG15*, ubiquitination detection is enhanced in breast cancer cells. Similarly, when the expression of the E2 ISG15 conjugating enzyme (*UBE2L6*) is reduced, the ISGylation levels decrease, while ubiquitination marks increase [36]. Another study showed that Lys 29 and Lys 48 from ubiquitin are substrates for ISG15, forming ubiquitin-ISG15 chains that are not degradable and affecting the binding of ubiquitin to its target proteins [37]. ISGylation maintains the stability of some proteins by inhibiting their polyubiquitination and degradation; these include pSTAT1, EMD, and YAP. Hence, pSTAT1 is ISGylated to stabilize its activity as a transcription factor [38]; the stability of EMD conferred by ISGylation promotes glucose metabolism in lung adenocarcinoma (LUAD) [31]; and the ISGylation of YAP confers stability to promote pro-tumor actions [32]. By contrast, an increase in ubiquitination levels was observed with ISG15 overexpression in HepG2 cells [39]. Some studies have shown that ISGylation can also promote the degradation of proteins, but the molecular details are not completely clear. Some examples are β-catenin [27, 28], FOXO3A, and PTEN [29, 30], which seem to present ISGylation-associated ubiquitination for their degradation in cancer contexts (Table 1).

### ISGylation is associated with lysosomal pathways in cancer

Autophagy is a catabolic process that delivers cellular components to lysosomes and organelles for macromolecule destruction. Not only is protein ISGylation related to the UPS but this modification has also been associated with lysosomal pathways. ISG15 overexpression in U251 glioma cells increases the ISGylation and autophagic degradation pathways [40]. ISGylation has been shown to inhibit exosome secretion, leading to lysosomal degradation of multivesicular bodies (MVBs) proteins [41]. Nevertheless, ISG15 enhances the stability of Ki-Ras, inhibiting its lysosomal degradation in breast cancer cells [42]. Furthermore, *ISG15* or *UBE2L6* depletion leads to increased autophagy in esophageal cancer cells, suggesting that ISGylation can inhibit autophagy [43]. Interestingly, type I IFN-induced ISGylation at residues Lys 117, 263, 265, and 266 of BECN1 was observed in 293T and HepG2 cell lines. The ISGylation of BECN1 inhibits autophagy, but the de-ISGylase USP18 facilitates autophagy and the degradation of EGFR by promoting the de-ISGylation of BECN1 [15].

### ISGylation modulates molecular interactions

Protein-protein interactions that form multiprotein complexes also seem to be affected by ISGylation modifications. Filamin B has been demonstrated to be a scaffold for Rac family small GTPase 1 (RAC1), MAPK/extracellular signal-regulated kinase (ERK) kinase kinase 1 (MEKK1), and MAPK kinase 4 (MKK4) proteins, which are part of IFN-α/β-induced JNK signaling to induce apoptosis. When filamin B is ISGylated, its scaffold functions are interrupted, decreasing JNK signaling and its actions [18]. Further, the scaffold protein IQGAP1 and the cytoskeletal protein, non-muscle myosin IIA (NMIIA), are targets of ISGylation in the breast cancer context. However, the ISGylation of these proteins has not been related to changes in protein stability, suggesting that the effects of this modification may affect protein-protein interactions [44–46].

### ISGylation modulates the activity of some proteins

Other molecular interactions affected by ISGylation may affect cellular processes. For example, TLS via DNA polymerase N is induced when the PCNA is monoubiquitinated and then ISGylated in response to DNA damage by ultraviolet (UV) light. As a result of PCNA ISGylation, the ubiquitination mark is removed, leading to the release of polymerase N from PCNA for TLS termination [24]. Furthermore, the 4EHP [messenger RNA (mRNA) 5’ cap structure-binding protein] is modified by HHARI-dependent ISGylation, increasing its binding to the cap and competing with the eukaryotic translation initiation factor 4E (eIF4E) translation initiation factor [21]. Further, when the E2-ubiquitin conjugation enzyme UBC13 is ISGylated, its activity decreases, affecting the ubiquitination process [47]. However, ISGylation enhances the activity of the CHIP and PARK, two E3 ubiquitin ligases that mark and induce the degradation of their substrates [19, 48].

Furthermore, proteins can display several changes due to their ISGylation. For example, the ISGylation of EMD confers stability but is also required for the interaction between EMD and pyruvate dehydrogenase E1 α subunit (PDHA) protein to inhibit aerobic oxidation [31]. Similarly, YAP ISGylation results in its stability, reducing its interaction with E3-ubiquitin ligase β-transducin repeat containing E3-ubiquitin protein ligase (βTrCP); however, this modification of YAP favors its activity promoting the transcription of genes, such as *PGLS* that encodes 6-phosphogluconolactonase (6PGL) of the pentose phosphate pathway (PPP). This glucose metabolism pathway promotes tumor growth in LUAD [32].

It has been proposed that genomic stability may be conferred through ISGylated proteins by mitigating DNA replication stress [49]. Moreover, the metabolic plasticity and mitophagy of pancreatic cancer (PC) stem cells also seem modulated by protein ISGylation. *ISG15* depletion reduces ISGylation in mitochondria, impairing mitophagy and reducing oxidative phosphorylation [50].

## Free ISG15: another face of ISG15

Whereas the ISGylation system promotes the covalent binding of ISG15 to its target proteins, USP18 is a deISGylase protein that removes ISG15 from its modified proteins, maintaining ISG15 in its free form (non-conjugated). Some studies have demonstrated that USP18^–/–^ models display enhanced ISGylation levels [9, 51–53]. By contrast, increased USP18 activity can enhance free ISG15 levels [10, 11]. Interestingly, ISG15 is a protein modifier and a cytokine-like protein, since non-conjugated/free ISG15 is secreted from some immunologic cells, such as lymphocytes and monocytes, and recognized by natural killer (NK) cells and CD3+ T cells [54–58]. These cells express an integrin receptor containing αL and β2 integrin subunits (LFA-1) integrin-type receptor. LFA-1 receptor recognition of ISG15 induces the secretion of IFN-α and interleukin-10 (IL-10) [59]. In the context of cancer, free ISG15 may also be secreted and may act as a potential factor in the microenvironment of malignant tumors [60, 61]. Furthermore, free ISG15 seems to have intracellular actions via protein-protein interactions, which are mentioned briefly in Table 2 and Table 3.

**Table 2:** Actions of free ISG15 in some cancer types

**Cancer type**	**Actions**	**Reference**
PDA	TAMS from patients with PDA exhibits a high *ISG15* expression. TAM secretes ISG15, increasing the phenotype of CSCs. Moreover, IFN-β promotes that CSCs also secrete ISG15	[62]
Melanoma	Soluble ISG15 is secreted to medium from melanoma cells, promoting E–cadherin expression on human dendritic cells	[63]
ESCC	Patients with ESCC have increased *ISG15* expression High levels of free ISG15 are found in the plasma of patients with ESCC compared with healthy patients	[64]
Breast cancer	Exogenous-free ISG15 reduces tumor growth in athymic mice by promoting NK cell infiltration Intracellular-free ISG15 promotes the expression of MHCI	[65]
OSCC	Free ISG15 interacts with Rac1-GDP, promoting cell migration in an ISGylation-independent manner. This event has been related to lymphatic metastasis of OSCC	[66]

PDA: pancreatic ductal adenocarcinoma; TAMs: tumor-associated macrophages; ESCC: esophageal squamous cell carcinoma; MHCI: major histocompatibility complex I; OSCC: oral squamous cell carcinoma; CSCs: cancer stem cells

**Table 3:** Effects of free ISG15 that may be relevant in a cancer context

**Effect**	**Description of free ISG15-associated effects**	**Reference**
Regulation of IFN signaling	In humans, JAK1-IFNAR2 interaction is disrupted by USP18, affecting IFN-α/β signaling Free ISG15 interacts with USP18, inhibiting its degradation by SKP2	[67]
Protein complex disassembly	Intracellular free ISG15 interrupts the USP18-SKP2 interaction promoting the stability of USP18	[68]
Regulation of E3-ubiquitin ligase activity	Free ISG15 binds to the E3-ubiquitin ligase NEDD4 to interrupt its interaction with the E2 biquitin-conjugating enzyme, decreasing ubiquitination	[69]
Participation in selective autophagy	LRRC25 inhibits the IFN-signaling by promoting lysosomal degradation of ISG15-associated RIG-1	[70]

JAK1-IFNAR2: janus kinase 1-IFN α and β receptor subunit 2; SKP2: S-phase kinase-associated protein 2; NEDD4: NEDD4 E3 ubiquitin protein ligase (also known as NEDD4-1 or RPF1, neural precursor cell expressed, developmentally down-regulated 4); LRRC25: leucine-rich repeat containing 25; RIG-1: RNA sensor RIG-1

## Deregulation of *ISG15* expression and its implications in cancer

**Figure 3:**
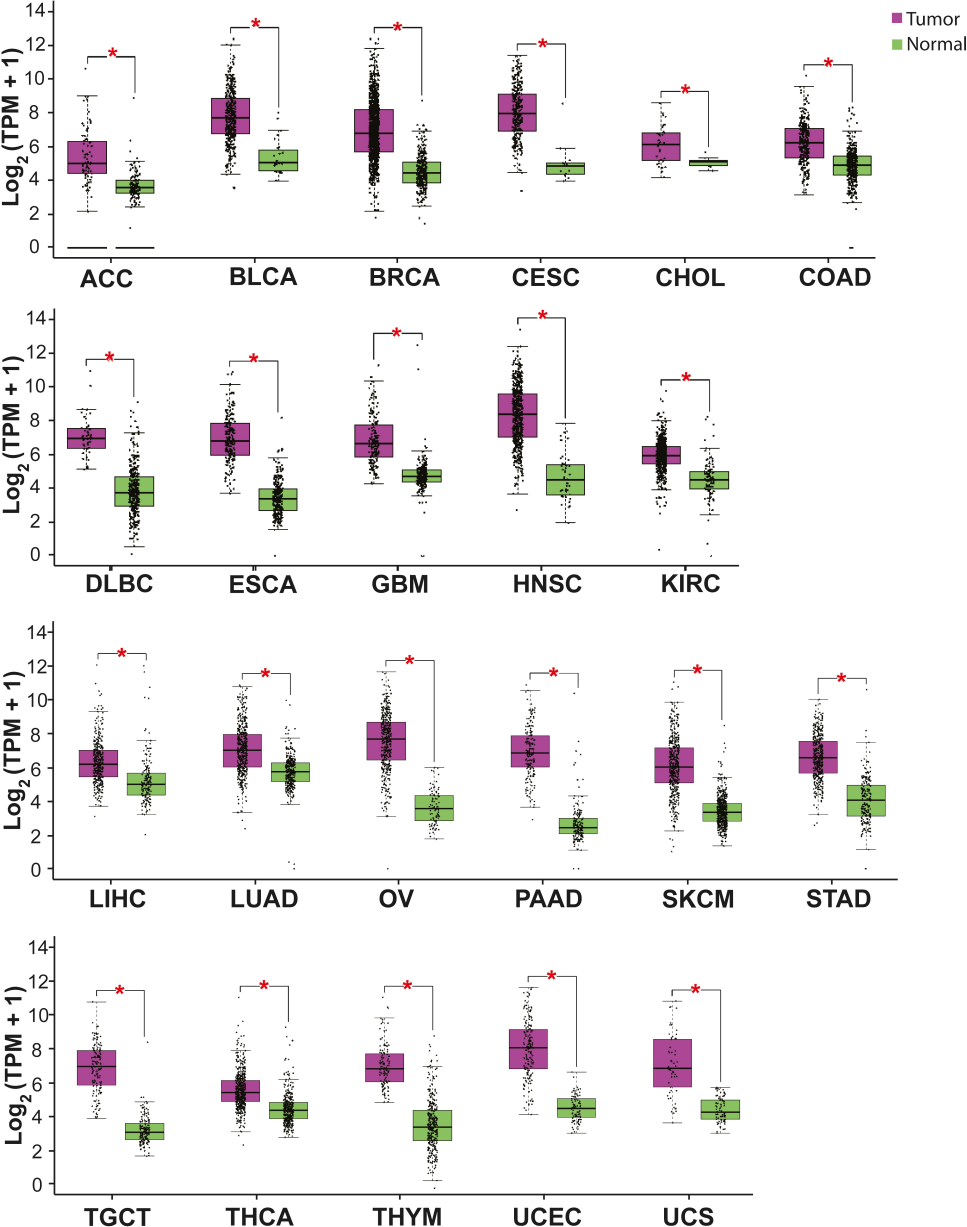
*ISG15* expression in some cancer types. The graph shows ISG15 expression in several malignant tumors (purple) compared with normal (healthy) tissue (green). The asterisk (*) indicates that the difference is statistically significant (*P* < 0.01). Graph analyzed using gene expression profiling interactive analysis (GEPIA, http://gepia.cancer-pku.cn/index.html). TPM: transcripts per kilobase of exon model per million mapped reads; ACC: adrenocortical carcinoma; BLCA: bladder urothelial carcinoma; BRCA: breast invasive carcinoma; CESC: cervical squamous cell carcinoma and endocervical adenocarcinoma; CHOL: cholangiocarcinoma; COAD: colon adenocarcinoma; DLBC: lymphoid neoplasm diffuse large B cell lymphoma; ESCA: esophageal carcinoma; GBM: glioblastoma multiforme; HNSC: head and neck squamous cell carcinoma; KIRC: kidney renal clear cell carcinoma; KIRP: kidney renal papillary cell carcinoma; LIHC: liver hepatocellular carcinoma; OV: ovarian serous cystadenocarcinoma; PAAD: pancreatic adenocarcinoma; SKCM: skin cutaneous melanoma; STAD: stomach adenocarcinoma; TGCT: testicular germ cell tumor; THCA: thyroid carcinoma; THYM: thymoma; UCEC: uterine corpus endometrial carcinoma; UCS: uterine carcinosarcoma

*ISG15* expression is increased in most cancer types. High levels of ISG15 mRNA have been detected by RNA-sequencing (RNA-seq) in several malignant neoplasias from patients’ samples (Figure 3) [71]. High levels of ISG15 protein have also been determined by immunochemistry, including breast, nasopharyngeal, and oral carcinomas [72–76]. These results indicate an upregulation of ISG15 in cancer, suggesting a pro-tumor role of ISG15.

Some studies have shown that the depletion of *ISG15* expression reduces the proliferation and migration of breast carcinoma-derived cells [42, 77]. *ISG15* expression is also deregulated in other cancer types developed in the central nervous system. For instance, glioblastoma displays high levels of ISG15, which are associated with poor survival [78]. *ISG15* upregulation also seems important for the ISGylation of essential proteins, such as OCT4, and the cell stemness of glioblastoma cells [34]. Nevertheless, ISG15-dependent antitumor activities, including the reduction of proliferation, tumor growth, and the induction of apoptosis, have been reported in some cancers, such as ovarian cancer and leukemia [79, 80]. For instance, reduced *ISG15* expression in ovarian cancer seems related to a poor prognosis for patients with this disease [81]. Therefore, the deregulation of *ISG15* expression may display pro- and anti-tumor activities depending on the cancer type.

The dual activities of ISG15 in cancer may be related to the crosstalk of ISG15 with other molecular pathways. For example, ISG15 is increased in the cervical cancer context, and ISG15 depletion inhibits proliferation and migration, suggesting its pro-tumor role [82]; however, ISG15 also displays antitumor activities in cervical cancer cells but in a p53-dependent manner [83]. Another example is *ISG15* upregulation in endometrial carcinoma to promote MYC proto-oncogene (MYC) signaling and changes in the DNA methylation profile, leading to pro-tumor actions and a poor prognosis [84].

Notably, the expression of the ISGylation enzymes and the USP18 deISGylase are critical in determining the relationship between ISGylation and free ISG15 levels and, consequently, their functions in cancer. Although type I IFNs α and β are the classical inductors of *ISG15* expression [2, 54–56, 85], IFN-γ also induces *ISG15* expression, depending on the cell type [86]. The ISGylation system (*UBA7*, *UBE2L6*, *HERC5*, and *TRIM25*) can also be regulated in response to type I IFNs [22, 83, 87–89]. Moreover, several molecular pathways can modulate *ISG15* expression in a cancer context, some of which are summarized in Table 4. Thus, protein ISGylation can be increased by several deregulated signaling pathways in cancer.

**Table 4:** Molecular pathways that modulate ISG15/ISGylation levels in a cancer context

**Molecular pathway**	**Elements related to the ISGylation system**	**Effect**	**Cellular context**	**Reference**
TNF-α, p38, and MAPK/JNK	*ISG15* *UBA7* *UBE2L6* Protein ISGylation	Up	A549 (lung cancer) and HSC4 (human OSCC) cell lines	[90, 91]
Androgens	*ISG15* Protein ISGylation	Down	Prostate cancer cells (LNCaP cell line)	[92]
Integrins (α5β1 and αV) through MRTF-A/SRF	*ISG15*	Up	MDA-MB-231 breast cancer cell line	[93]
KLF9	*ISG15*	Down	HT29 CRC cells and a mouse model of CRC	[94]
RA	*ISG15* *UBA7* *USP18*	Up	RA-sensitive leukemia cells	[95]
CYP1B1	*ISG15* *HERC5*	Down	Hela cells	[28]
UV	*ISG15* *UBEA7* *UBE2L6* ISGylation of PCNA	Up	Hela cells	[24]
DNA damage	*ISG15* *UBA7* *UBE2L6* *TRIM25*	Up	HEK293T, A549, and H1299 cell lines	[25]
Hypoxia	*ISG15* *ISGylation enzymes* *USP18* ISGylation of HIF-1α	Up	Human 769-P, Caki-1, and 293T renal cell lines	[26]
miR-138	*ISG15* mRNA	Down	Oral squamous carcinoma cells (CAL27 and SCC15 cells)	[96]
miR-370	*ISG15* mRNA	Down	Hepatocellular carcinoma cells	[97]
Inhibition of *SOCS3* via miR-2909	*STAT1* *ISG15* Protein ISGylation	Up	LNCaP prostate cancer cell line	[92]
BAG3	*ISG15* mRNA ISG15 protein	Down (mRNA) Up (protein)	PDACs	[98]
SOCS1	*STAT1* *UBE2L6* Protein ISGylation	Down	iPSCs	[99]
WBSCR22	*ISG15*	Down	PC cells	[100]
Curcumin	Protein ISGylation	Down	MCF10A (human mammary tissue) and A549 (lung cancer) cells	[101]
KLF12	*ISG15*	Down	Cisplatin-resistant ovarian cancer cells	[81]

TNF-α: tumor necrosis factor-α; MRTF-A: myocardin-related transcription factor A; SRF: serum response factor; KLF9: KLF transcription factor 9; RA: retinoic acid; CYP1B1: cytochrome P450 family 1 subfamily B member 1; miR-138: microRNA-138; *SOCS3*: suppressor of cytokine signaling 3; BAG3: BAG cochaperone 3; PDACs: PDA cells; iPSCs: induced pluripotent stem cells; WBSCR22: BUD23 rRNA methyltransferase and ribosome maturation factor; KLF12: KLF transcription factor 12; CRC: colorectal cancer

Further, some components of the ISGylation system, such as UBCH8 and TRIM25, can be shared with the ubiquitination system. *UBE2L6* has been demonstrated to be the primary target gene for IFN-α and IFN-β in A549 lung epithelial cells, HepG2 hepatoma cells, and NK-92 cells [102]. *UBE2L6* encodes UBCH8, which is also implicated in ubiquitination reactions for protein degradation via the UPS [103]. Histone deacetylation inhibitors (e.g., LBH589) increase UBCH8 levels, favoring the degradation of a mutated form of fms related receptor tyrosine kinase 3 (FLT3) associated with acute myeloid leukemia [104]. These data suggest that alterations in other posttranslational modifications may also affect protein ISGylation in the cancer context. TRIM25 is also an E3-ubiquitin ligase associated with the ubiquitination and degradation of some tumor suppressors. However, TRIM25 is one of more than 600 E3 ligases in the ubiquitination system [105, 106]. It is unclear whether there is an interplay between ubiquitination and ISGylation mediated by TRIM25.

## ISG15 as a potential therapeutic target for cancer

The high levels of ISG15 in several cancer types suggest the potential of ISG15 as a biomarker. Moreover, some cancer therapies, such as chemotherapy, radiotherapy, and targeted therapy, seem to be affected when ISG15 levels are deregulated, suggesting that ISG15 may be implicated in the response to cancer therapies (Table 5). In addition, the ISG15 protein has been considered a novel tumor-associated antigen to generate a Listeria-based vaccine targeting ISG15 (Lm-LLO-ISG15) [107, 108]. Nevertheless, more studies are required to understand the role of ISG15 in cancer therapies. The association between ISG15 and cancer therapies is summarized in Table 5.

**Table 5:** Interplay between ISG15/ISGylation and cancer therapy

**Therapy type**	**Cancer cells**	**The described role of ISG15 in cancer therapy**	**Reference**
Chemotherapy	A549 lung cancer cells	Resistance to cisplatin is observed due to the silencing of *ISG15* The reparation of cisplatin-damaged DNA in A549 cells reduces *ISG15* expression	[109]
Chemotherapy and targeted therapy	Ovarian cancer cells	Wild-type ISG15 overexpression (but not mutant ISG15 that is incapable of ISGylation) decreases ABCC2 protein levels, sensitizing resistant ovarian cancer cells to cisplatin	[110]
	SFT	The expression of CSC-related genes is decreased by *ISG15* downregulation, resulting in increased cell death in 3D cultures after doxorubicin, pazopanib, or trabectedin treatment	[111]
Chemotherapy and radiation	NPC cells	*In vivo* tumorigenicity and resistance to radiation and DDP by ISG15 overexpression	[74]
Radiotherapy	Chronic myeloid leukemia and colorectal carcinoma	Cytokines and antigen presentation-associated proteins can be the target of ISGylation. Hence, the downregulation of *USP18* enhances the response of CTLs, and cancer cells can become more susceptible to radiotherapy	[112]
Immunotherapy	CRC	Lm-LLO-ISG15 in an immunocompetent CRC murine model generates an anti-tumor response	[107]
	RCC	Lm-LLO-ISG15 vaccine in subcutaneous and orthotopic RCC mouse models results in adequate CTL-based immunotherapy, generating anti-tumor activity.	[108]
Other therapies	Cervical cancer, leukemia, and myeloma	The loss of NF-κB signaling causes ISG15 expression-induced apoptosis Clioquinol and mefloquine treatments induce high levels of ISG15	[80]

SFT: solitary fibrous tumor; ABCC2: ATP binding cassette subfamily C member 2; 3D: three dimensions; NPC: nasopharyngeal carcinoma; DDP: cisplatin; RCC: Renal cell carcinoma; CTLs: cytotoxic T lymphocytes; NF-κB: nuclear factor-κB

In summary, protein ISGylation is a posttranslational modification implicated in malignant neoplasm progression. ISGylation can modulate protein stability, either positively or negatively, by promoting or inhibiting ISGylated protein degradation via the UPS or lysosomes. Furthermore, ISGylation can modulate molecular interactions by generating or disassembling protein complexes. The mechanisms, targets, and functional consequences of ISGylation seem to be defined by cancer type. Several proteins have been identified as ISGylation targets, and the Lys residues where this modification occurs have only been reported in some of them. Thus, identifying and characterizing new ISGylation target proteins and exploring the molecular bases of ISGylation and its functional repercussions are still necessary. Protein ISGylation levels and the ISGylated protein types can affect the response to chemotherapeutic treatments; studies in more depth are required to understand the role of ISG15/ISGylation in cancer therapies. Moreover, it is important to consider that ISGylation levels are related to the deregulation of ISG15, ISGylation system enzymes, and USP18 de-ISGylase expression in cancer. These elements implicated in the modulation of ISGylation can also be helpful for the novel design of therapeutic strategies, but more investigation is required.

## Conclusions

ISGylation is a key posttranslational modification in malignant neoplasms, with implications for their progression, affecting adaptation in the tumor microenvironment and the ability to respond to cancer therapies. In most cancer types, protein ISGylation seems to be related to a pro-tumor effect, but its interplay with other molecular pathways depending on the cell type may determine the actions of this modification in promoting or reducing malignant neoplasm progression. Thus, ISG15/ISGylation can be over- or down-regulated depending on the type of cancer, and the identification of ISGylated proteins will help in understanding the novel molecular pathways associated with ISG15. Thus, protein ISGylation emerges as a central factor in cancer, which demands further investigation. Novel findings about the molecular mechanisms of ISG15 in tumorigenesis may be useful in preventing, treating, and controlling cancer.

## Abbreviations

4EHP: eukaryotic translation initiation factor 4E homologous protein
ATP: adenosine triphosphate
BECN1: beclin 1
EMD: skeletal protein emerin
FOXO3A: forkhead box O3A
HERC5: HECT and RLD domain containing E3 ubiquitin protein ligase 5
HHARI: ariadne RBR E3 ubiquitin protein ligase 1
IFN: interferon
ISG15: interferon-stimulated gene 15
JNK: c-Jun N-terminal kinase
Lm-LLO-ISG15: Listeria-based vaccine targeting interferon-stimulated gene 15
Lys: lysine
MAPK: mitogen-activated protein kinase
mRNA: messenger RNA
NK: natural killer
OCT4: POU class 5 homeobox 1 (also known as POU5F1)
p53: phosphoprotein 53
PARK: parkin
PCNA: proliferating cell nuclear antigen
pSTAT1: phosphorylated signal transducer and activator of transcription 1
PTEN: phosphatase and tensin homolog
TLS: translesion DNA synthesis
TRIM25: tripartite motif containing 25
UBA7: ubiquitin like modifier activating enzyme 7
UBCH8: ubiquitin-conjugating enzyme E2 L6
UBE1L: E1-activating enzyme
UBE2L6: E2 ISG15 conjugating enzyme
UBL: ubiquitin-like
UPS: ubiquitin-proteasome system
USP18: ubiquitin-specific peptidase 18
YAP: Yes-associated protein

## Declarations

### Copyright

© The Author(s) 2023.
